# Microbiota Dysbiosis in *Mytilus chilensis* Is Induced by Hypoxia, Leading to Molecular and Functional Consequences

**DOI:** 10.3390/microorganisms13040825

**Published:** 2025-04-05

**Authors:** Milton Montúfar-Romero, Diego Valenzuela-Miranda, Valentina Valenzuela-Muñoz, María F. Morales-Rivera, Cristian Gallardo-Escárate

**Affiliations:** 1 Interdisciplinary Center for Aquaculture Research (INCAR), Universidad de Concepción, P.O. Box 160-C, Concepción 4030000, Chile; mba.ing.ac.milton.montufar@gmail.com (M.M.-R.); valevalenzuela@udec.cl (V.V.-M.); marimoralesr@udec.cl (M.F.M.-R.); 2 Instituto Público de Investigación de Acuicultura y Pesca (IPIAP), Guayaquil 090314, Ecuador; 3 Centro de Biotecnología, Universidad de Concepción, P.O. Box 160-C, Concepción 4030000, Chile; 4 Center for Oceanographic Research COPAS COASTAL, Universidad de Concepción, Concepción 4070409, Chile

**Keywords:** gills, digestive gland, 16s rRNA sequencing, facultative anaerobes, metabolic efficiency, aquaculture, microbial dysbiosis

## Abstract

Bivalve microbiota play a vital role in host health, supporting nutrient processing, immunity, and disease resistance. However, the increasing hypoxia in Chilean coastal waters, caused by climate change and eutrophication, threatens to disrupt this microbial balance, potentially promoting pathogens and impairing essential functions. *Mytilus chilensis* is vulnerable to hypoxia-reoxygenation cycles, yet the effects on its microbiota remain poorly understood. This study investigates the impact of hypoxia on the structure and functional potential of the microbial communities residing in the gills and digestive glands of *M. chilensis*. Employing full-length 16S rRNA gene sequencing, we explored hypoxia’s effects on microbial diversity and functional capacity. Our results revealed significant alterations in the microbial composition, with a shift towards facultative anaerobes thriving in low oxygen environments. Notably, there was a decrease in dominant bacterial taxa such as Rhodobacterales, while opportunistic pathogens such as *Vibrio* and *Aeromonas* exhibited increased abundance. Functional analysis indicated a decline in critical microbial functions associated with nutrient metabolism and immune support, potentially jeopardizing the health and survival of the host. This study sheds light on the intricate interactions between host-associated microbiota and environmental stressors, underlining the importance of managing the microbiota in the face of climate change and aquaculture practices.

## 1. Introduction

Dissolved oxygen (DO) levels are critical for the survival and health of marine organisms [1]. In coastal marine ecosystems, reference thresholds have been established to consider the biological and ecological effects of dissolved oxygen levels in the ocean [2,3,4,5,6,7,8]. Normoxia is defined as the condition in which DO levels range between 9.0 and 3.0 mg L^−1^, while hypoxia occurs when DO levels fall between 2.0 and 0.1 mg L^−1^ [2,3,4,5,6,7,8]. Hypoxia on the south-central coast of Chile is a pressing issue severely affecting the region’s bivalve mollusks, with potentially severe consequences for the marine ecosystem [9,10,11,12,13]. Long-term research reveals that hypoxic zones occupy substantial portions of the water column during upwelling seasons, with an intensifying trend linked to climate change and altered oceanographic processes [14]. These trends portend a grim outlook for marine organisms reliant on stable oxygen availability [14].

Hypoxia-induced stress can severely impact bivalves, ranging from physiological to molecular levels [15,16]. Physiologically, this stress can increase clearance and respiration rates and reduce food intake, potentially resulting in stunted growth in these organisms [15,17]. Additionally, hypoxic stress can negatively affect the immune system of bivalves, increasing their susceptibility to pathogen infections [18,19]. At the cellular level, hypoxia can trigger autophagy, increase oxidative stress, and reduce cell viability [20]. Molecularly, prolonged hypoxia can affect protein metabolism, inflammation-related genes, and programmed cell death [20,21]. In extreme cases, hypoxic stress can lead to mass mortality and stranding of bivalves [10].

Within bivalve mollusks, the microbiota is characterized as a dynamic and interactive consortium of microorganisms, encompassing bacteria, archaea, viruses, fungi, and protozoa, which establish residence within diverse biological niches, including the mantle, gills, digestive gland, hemolymph, gonad, byssus gland, adductor muscle, and mucus. This engagement results in intricate symbiotic relationships with the host, modulating and executing critical physiological processes [22,23,24,25,26,27,28,29,30]. The microbiota associated with the host plays a crucial role in animal health by providing vital functions such as disease protection and nutrient processing [15,22]. Moreover, microbiota composition influences the host’s physiology, stress tolerance, and fitness [31]. The microbiota is considered an organ that regulates host metabolism and is essential for maintaining a healthy balance in the host immune system due to its relationship with specific diseases [32,33,34,35]. Recent studies have shown that a diverse and balanced microbiota can indicate better metabolic health [36]. Greater microbiota diversity is associated with improved lipid profiles, lower levels of pro-inflammatory cytokines, and higher levels of anti-inflammatory cytokines [36]. Furthermore, bivalve mollusks have also shown correlations between microbiota diversity, enzyme activity, and genetic pathways related to metabolism and health [36,37,38].

Intestinal microbiota significantly influence the host’s physiology, reproduction, development, energy balance, behavior, and life history [39]. The intestinal microbiota of bivalve mollusks play an essential role in their health and nutrition [40]. The diversity and abundance of microorganisms in their digestive tract assist in food digestion, strengthen the immune system, and may influence their growth and development [40,41,42]. However, stress-induced alterations in microbial communities, such as those caused by hypoxia, may increase disease risk and compromise bivalve health [43,44]. For example, the proliferation of opportunistic pathogens, including those from the *Vibrio* and *Arcobacter* genera, could significantly increase the host’s susceptibility to diseases, contributing to increased mortality [45]. Furthermore, fluctuations in the external environment, such as abiotic factors, can alter the structure, species richness, and diversity of intestinal microbiota [15].

*Mytilus chilensis* is a bivalve species of ecological and economic importance in the coastal waters of the Los Lagos Region in Chile [46,47]. Climate change, manifested in declining oxygen levels in the water, induces systematic changes in bivalve mollusks and their bacterial symbionts [15,48,49]. Economically valuable bivalve species, including *M. chilensis*, are increasingly exposed to hypoxic conditions, threatening their viability and sustainability [50,51]. The aquaculture industry in southern Chile, heavily relies on seed collection from the Reloncaví Fjord and grow out operations around Chiloé Island, faces recurrent hypoxia episodes exacerbated by seasonal upwelling and anthropogenic eutrophication [52,53].

The Reloncaví system, comprising the Reloncaví Fjord and Reloncaví Sound, is particularly vulnerable to hypoxia due to the influx of suspended allochthonous organic matter from rivers, especially during late winter and early spring [53,54,55]. This period is marked by glacial meltwater contributions, dominating precipitation-driven runoff [53,54,55]. Major riverine inputs, including the Puelo, Petrohué, and Cochamó Rivers, deliver substantial organic material, fueling microbial decomposition and oxygen consumption [56,57]. Recent risk assessments identify the Reloncaví estuarine system as a hotspot for high inorganic nutrient concentrations, intense phytoplankton blooms, and elevated chlorophyll levels, particularly in late winter (August–September) [58,59,60]. These conditions, driven by eutrophication from intensive salmon aquaculture, promote the formation of low dissolved oxygen water (LDOW) zones, where stratification and particulate organic matter deposition exacerbate oxygen depletion [60]. The prolonged water residence times in the Reloncaví system, coupled with high biological oxygen demand—including phytoplankton respiration and bacterial remineralization of organic material—underscore the urgent need to investigate hypoxia’s impact on *M. chilensis*, particularly at the microbiota level [53,60,61,62,63].

Therefore, our objective is to understand the influence of hypoxia on the intestinal and gill microbiota of the native mussel *M. chilensis*. Specifically, a comparative evaluation of the bacterial communities in the intestine and gills of *M. chilensis* exposed to hypoxia was conducted using 16S rRNA sequencing with nanopore technology. Our study is the first to investigate the effects of hypoxia on *M. chilensis* from a hologenome concept. This knowledge could enhance our understanding of host-specific microbiotas and their roles in supporting host ecology. Additionally, it can help elucidate the physiological responses of *M. chilensis* to hypoxia and infer potential health and disease changes that may arise from future stress factors.

## 2. Materials and Methods

### 2.1. Experimental Design (Mussel Acclimatization, Hypoxia Challenge, and Sampling for Microbiological Analysis)

Understanding marine bivalves’ physiological and microbiological responses to hypoxic stress is crucial for assessing the impacts of climate change and coastal eutrophication on aquaculture species. While numerous studies have examined short-term hypoxia (15 min to 36 h) and subsequent reoxygenation cycles (10 min to 24 h) [20,21,64,65,66,67,68,69,70,71], research on prolonged hypoxic exposure remains limited despite reports of hypoxia lasting up to six days [21,60] in some coastal environments and 25 days [72,73,74] in others. This study was designed to simulate long-term progressive hypoxia rather than replicate singular hypoxic events, which naturally persist for at least two days in Chilean coastal ecosystems [14]. To approximate future oxygen variability, the experimental design was based on tidal coefficients obtained from publicly available tidal data (https://tablademareas.com accessed on 7 March 2022), reflecting fluctuations in tidal amplitude within the study area over approximately 10 days. The southern Chilean fjords, characterized by their complex coastal morphology and low water exchange rates (e.g., a residence time of approximately 98 days in the Reloncaví fjord), provide an ideal setting to investigate the impacts of prolonged hypoxia [53,75,76].

This study employed a controlled experimental approach to assess the systemic effects of long-term hypoxia and reoxygenation on *M. chilensis*, with a particular emphasis on microbiological shifts in gill and digestive gland tissues. The experimental design simulated environmental conditions projected under climate change and eutrophication scenarios.

Blue mussels (*M. chilensis*) utilized in this study were sourced in April 2022 from the experimental laboratory at the Marine Biological Station of the Universidad de Concepción, Chile. The experiment spanned 50 days and consisted of alternating hypoxic (dissolved oxygen concentrations of 2.0 mg/L) and normoxic (dissolved oxygen concentrations of 7.2 ± 0.2 mg/L) phases.

From an initial pool of 480 individuals, 36 mussels were selected and distributed into three experimental replicates (*n* = 12 mussels per replicate). The duration of each hypoxic exposure was determined based on methodologies established in prior studies [77]. The experimental design incorporated a structured sampling regime: Gill and digestive gland samples of three mussels were collected under hypoxic conditions at day 10 (*n* = 9 total mussels across replicates); gill and digestive gland samples of three mussels were taken following reoxygenation (normoxic conditions) at day 20 (*n* = 9); gill and digestive gland samples of three mussels were collected following reoxygenation at day 40 (*n* = 9); and gill and digestive gland samples of three mussels were sampled under hypoxic conditions at day 50 (*n* = 9). In total, 18 gill and digestive gland mussel samples were under hypoxia, and 18 were under reoxygenation. In the present experimental design, a control group was not included due to the primary focus on direct comparisons between hypoxic and reoxygenation conditions. Furthermore, a singular sampling event on days 10 and 20 would have critically compromised the study’s temporal resolution, precluding the detection of progressive effects or prolonged microbial shifts. The serial sampling at days 40 and 50 provided a detailed perspective on the long-term, cumulative effects of hypoxia and reoxygenation, essential for evaluating the impacts of oxygen variability on bivalves under climate change and eutrophication scenarios. This approach is fundamental for developing effective management and conservation strategies for *M. chilensis* in the face of evolving environmental conditions.

Before the hypoxia challenge, mussels were acclimatized for 38 days in filtered seawater (12.5 ± 0.94 °C) under continuous flow, aeration, and feeding. Following acclimatization, dissolved oxygen concentrations within the recirculation system were monitored daily and adjusted using nitrogen gas injection to maintain the target hypoxic level of 2.0 mg/L.

Gill and digestive gland tissues were selected due to their critical roles in bivalve physiology. Gills, as the primary interface between the organism and its environment, are central to respiration and filter feeding [78,79]. Given their constant exposure to ambient conditions, gills are particularly susceptible to hypoxia-induced physiological stress. Consequently, microbial changes within gill tissues were investigated as potential biomarkers of hypoxia-induced stress [80].

In addition to gills, the digestive gland was analyzed due to its multifunctional role in nutrient assimilation, metabolic regulation, and immune response [81,82,83,84]. Under hypoxic conditions, bivalves often exhibit valve closure, significantly reducing filtration and respiration rates [85,86]. Since hypoxia impacts filtration-dependent nutrient processing and metabolic activity, the digestive gland microbiota shifts were examined to elucidate the broader physiological consequences of hypoxia-induced dysbiosis. This study aimed to identify shared perturbation patterns and assess the physiological implications of hypoxia exposure in *M. chilensis* by examining microbial dynamics in both gill and digestive gland tissues.

**Figure 1:**
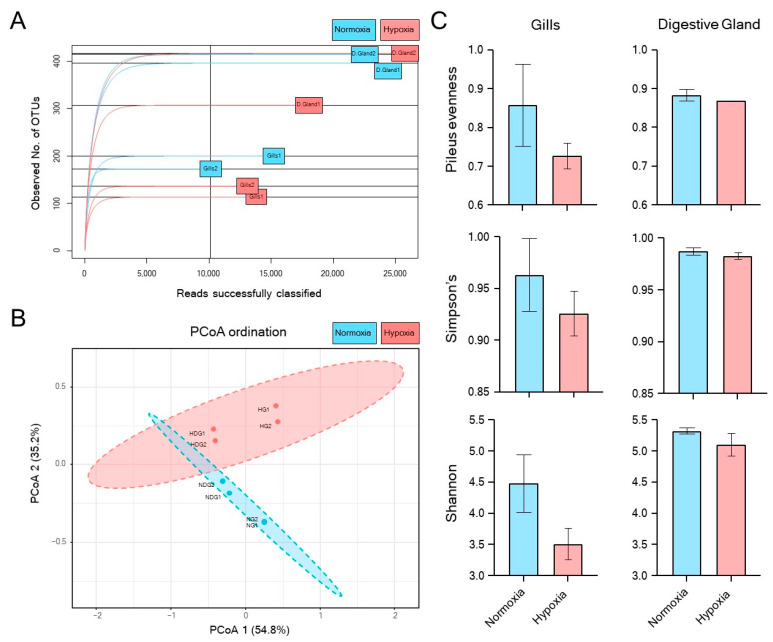
Alpha and beta-diversity analysis for *M. chilensis* microbiota exposed to normoxia (blue) and hypoxia (red). The figure shows the rarefaction curves for all the samples. The digestive gland is represented as D. Gland (**A**). In the principal coordinate analysis (PCoA), the digestive gland is represented as NDG (normoxia) and HDG (hypoxia), while the gills are denoted as NG (normoxia) and HG (hypoxia) (**B**). Different alpha diversity indexes were estimated for gills and digestive glands under the experimental conditions (**C**).

**Figure 2:**
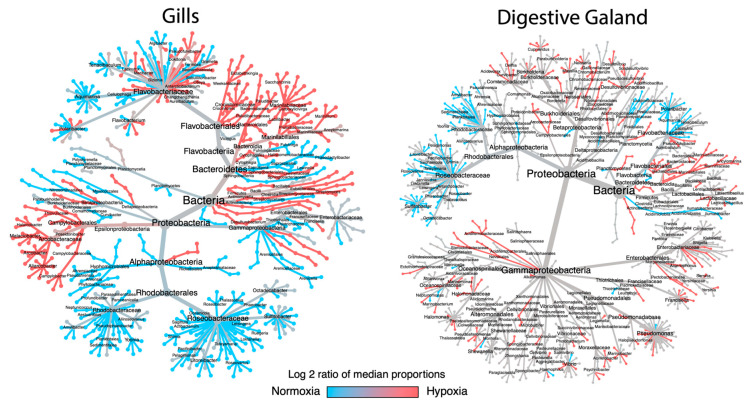
Heat tree analysis evidencing the taxonomical changes in the microbiota of gills and digestive gland of *M. chilensis* exposed to different levels of oxygenation. The color of the branches represents the log2 ratio of median proportions between normoxia (blue) and hypoxia (red).

**Figure 3:**
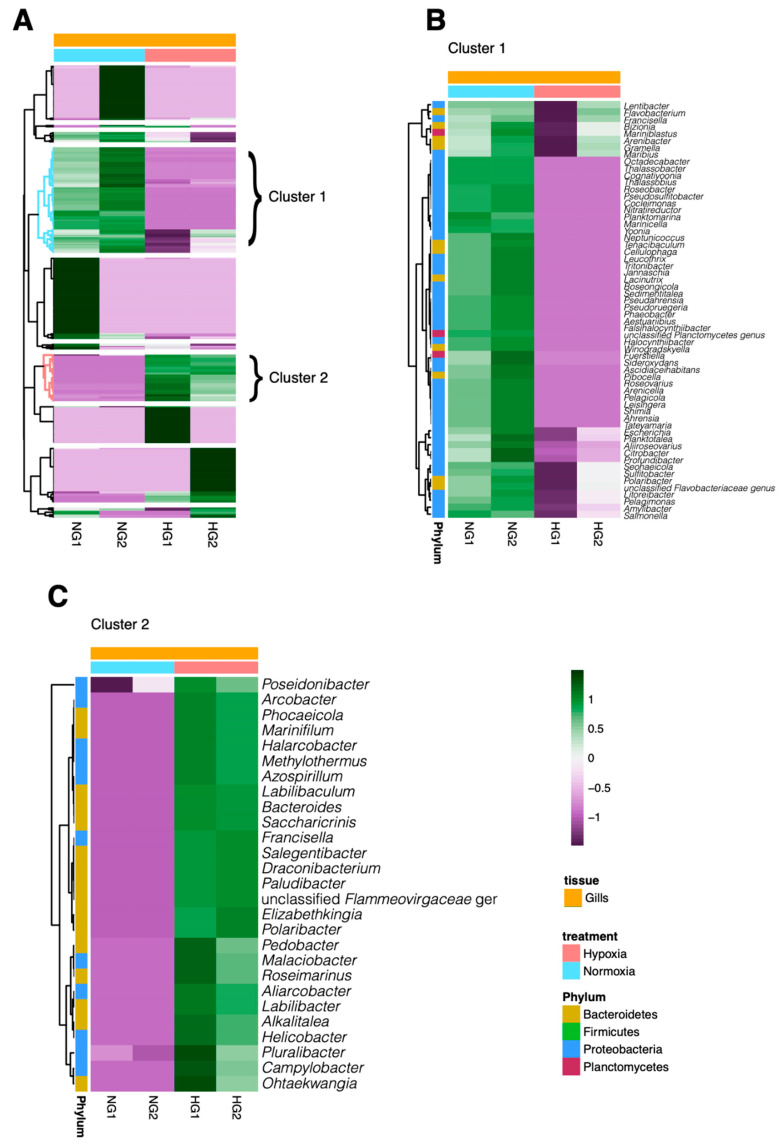
Heatmaps showing the relative abundance of various bacterial genera in the microbiota of gills (**A**) of *M. chilensis* exposed to normoxia (blue) and hypoxia (red). For each condition, two clusters were identified. “Cluster 1” includes genera that exhibited decreased abundance in the gills of mussels exposed to hypoxia (**B**). In contrast, “Cluster 2” comprises genera that increased their abundance in hypoxic conditions in the gills (**C**).

**Figure 4:**
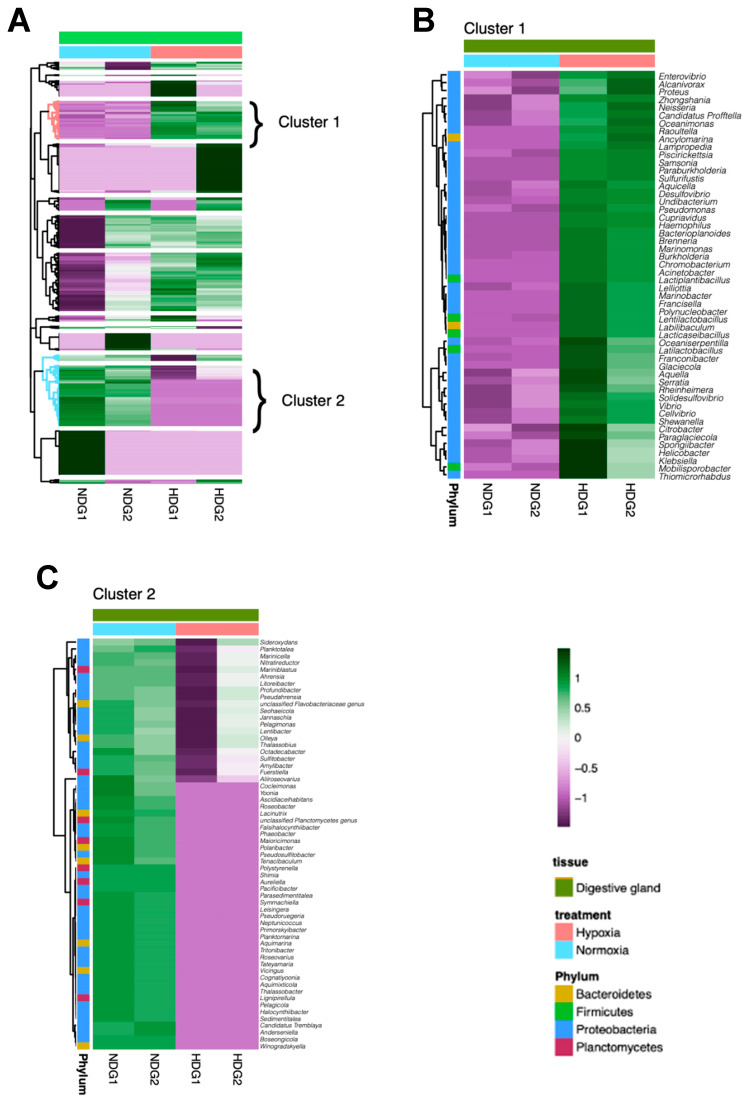
Heatmaps illustrating the relative abundance of different bacterial genera in the microbiota of the digestive gland (**A**) of *M. chilensis* exposed to normoxia (blue) and hypoxia (red). Two distinct clusters were identified for each condition. “Cluster 1” consists of genera that decreased in abundance in the digestive glands of mussels exposed to hypoxia (**B**), while “Cluster 2” includes genera that increased in abundance under hypoxic conditions (**C**).

**Figure 5:**
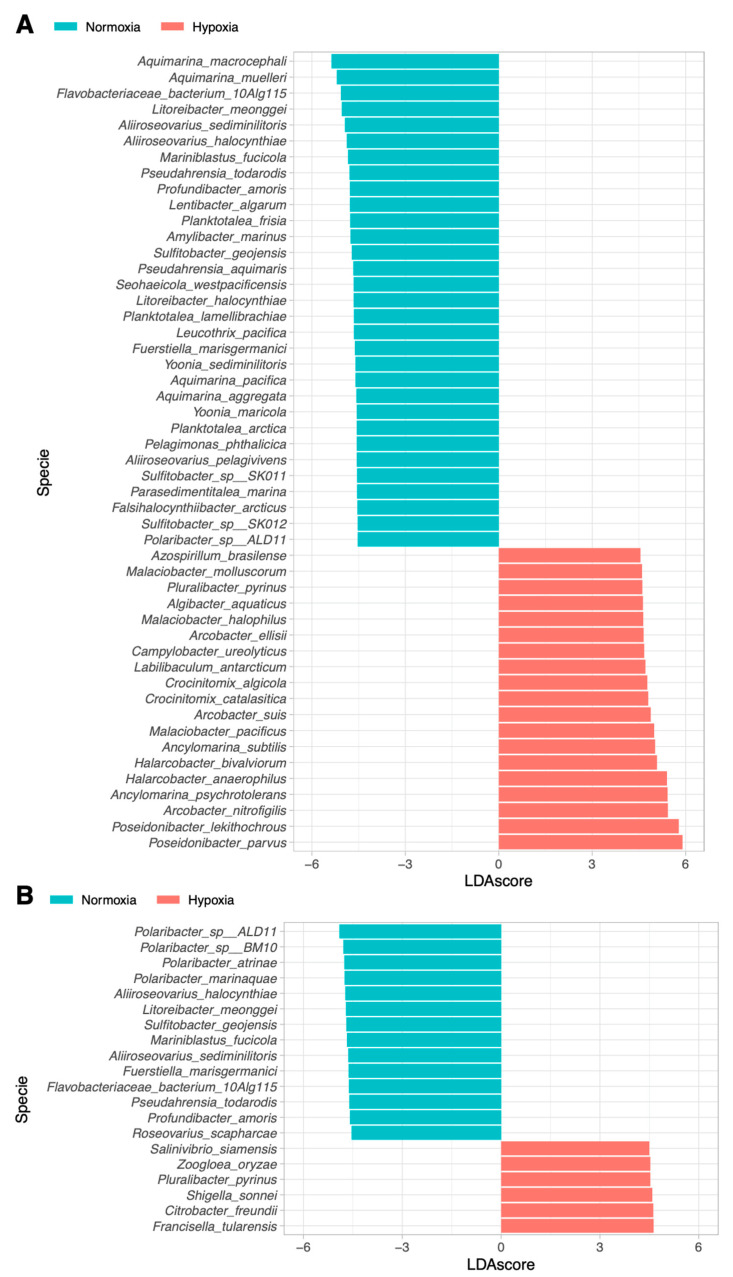
Linear Discriminant Analysis (LDA) scores of differentially abundant species in mussels exposed to normoxia and hypoxia conditions for gills (**A**) and digestive gland (**B**). Species with blue bars were significantly abundant in normoxia conditions, while the red ones were more abundant in hypoxic conditions.

To reduce inter-individual variability in the microbiota associated with gill and digestive gland tissues, samples from three mussels were pooled to create a single biological replicate, with each sequencing sample representing nine pooled individuals [87]. This pooling strategy, utilizing nine individuals per sequencing sample, was implemented to enhance the detection of consistent microbial patterns while optimizing sequencing resources. Figure 1A,B, 3, 4, and 7 present microbiota composition from pooled samples at control time points (days 20 and 40, *n* = 9 mussels each) and hypoxic phases (days 10 and 50, *n* = 9 mussels each). Conversely, Figure 1C, 2, 5, and 6 display microbiota composition from combined normoxic samples at days 20 and 40 (*n* = 18 total mussels) and combined hypoxic samples at days 10 and 50 (*n* = 18 total mussels).

The samples were preserved in molecular-grade ethanol, transported at 4 °C, and stored at −80 °C until further processing for microbiological analysis.

### 2.2. DNA Isolation and 16S Amplification

Total bacterial DNA was isolated from homogenized gill and digestive gland tissues of mussels using the phenol-chloroform extraction method.

Gill and digestive gland tissues (20–30 mg) were collected from three mussels per group (nine per treatment), thawed, washed, minced, and vortexed to facilitate homogenization using ceramic beads. Each sample was then mixed with 1 mL of lysis buffer (10 mM Tris-HCl, 400 mM NaCl, 100 mM EDTA, 0.4% SDS, and 100 μg/mL Proteinase K, pH 8.0) and incubated at 37 °C for 2 h under constant agitation to ensure complete lysis [88].

After incubation, 1 volume of phenol-chloroform was added to each sample, followed by centrifugation at 12,000 rpm for 5 min at room temperature. The aqueous phase was carefully collected, and an equal volume of chloroform was added, followed by an additional centrifugation at 12,000 rpm for 5 min. The resulting aqueous phase was mixed with molecular grade absolute ethanol and transferred to a DNeasy Blood and Tissue column (Qiagen, Germantown, MD, USA) to continue purification following the manufacturer’s protocol.

The quality and purity of the extracted DNA were assessed using a Nanodrop One spectrophotometer (Thermo Scientific, Waltham, MA, USA), and its integrity was verified through electrophoresis in a 1% agarose gel prepared in TAE buffer (Tris-Acetic Acid-EDTA). DNA concentration was further quantified by fluorescence using a Qubit 4 fluorometer (Thermo Scientific, Waltham, MA, USA) with the dsDNA BR Assay Kit (Thermo Scientific, Waltham, MA, USA).

For 16S rRNA gene amplification, the isolated DNA was diluted to a concentration of 50 ng/μL and used as a template in a 25 μL PCR reaction containing LongAmp Taq DNA polymerase (New England Biolabs, MA, USA) and universal 16S primers: 27F (5′-AGAGTTTGATCCTGGCTCAG-3′) and 1492R (5′-GGTTACCTTGTTACGACTT-3′) [88]. The thermal cycling conditions included an initial denaturation step at 95 °C for 1 min, followed by 25 cycles of 95 °C for 20 s, 56 °C for 30 s, and 65 °C for 2 min, with a final extension at 65 °C for 5 min. The resulting 16S rRNA PCR amplicons were confirmed by electrophoresis in a 1.2% agarose gel prepared in TAE buffer.

### 2.3. Library Preparation and Nanopore Sequencing

Nanopore sequencing is an advanced technique for characterizing microbial communities by sequencing the 16S rRNA gene amplicon [89]. Following the PCR amplification of the 16S rRNA gene, the resulting amplicons were pooled according to the experimental groups and purified using Agencourt AMPure XP beads (Beckman Coulter, Brea, CA, USA) to remove primer dimers and nonspecific amplification products. The purified amplicons were then quantified using a Qubit 4 fluorometer (Thermo Scientific, Waltham, MA, USA) to ensure the appropriate library concentration for sequencing.

Library preparation was conducted using the 16S Barcoding Kit (SQK-16S024, Oxford Nanopore Technologies, Oxford, UK) following the manufacturer’s protocol. The amplicons were barcoded through a PCR reaction using LongAmp Taq polymerase (New England Biolabs, Ipswich, MA, USA) and purified according to the supplier’s instructions.

The quality and size distribution of the prepared libraries were evaluated using the 2200 TapeStation system (Agilent, Santa Clara, CA, USA) with DNA ScreenTape (Agilent, CA, USA). This ensured that the amplicons were within the expected size range and minimized the presence of adapter dimers or residual primers. The final library concentration was determined using the Qubit 4 fluorometer with the High Sensitivity D5000 ScreenTape (Agilent, Santa Clara, CA, USA). A mock microbial community (ZymoBiomics Microbial Community Standard, Zymo Research, Irvine, CA, USA) was included in the analysis as a quality control standard to ensure accuracy and mitigate potential batch effects.

According to the manufacturer’s guidelines, libraries were pooled at equimolar concentrations for multiplexing and loaded onto a Spot-ON Flow Cell for sequencing using the MinION platform (Oxford Nanopore Technologies, Oxford, UK). The sequencing efficiency and run quality were monitored in real-time using the MinKNOW software (Oxford Nanopore Technologies) version: 5.8.12, enabling comprehensive and high-resolution microbial community profiling.

### 2.4. Data Processing and Taxonomic Assignment

A rigorous data processing pipeline was applied to the nanopore sequencing reads to ensure robust and reliable results. First, base-calling was performed using the Guppy software (version 6.3.2), which ensured high-quality nucleotide base identification. Subsequently, a strict quality filter (Q-score ≥ 7) was applied to remove low-quality reads, thereby maintaining the resulting dataset’s integrity.

The resulting FASTQ files were processed using Porechop to remove adapter sequences and to demultiplex the reads, assigning them to their respective samples [90]. The demultiplexed reads were then used as input for taxonomic classification through the Emu algorithm, specifically designed to annotate full-length 16S rRNA sequences generated from nanopore sequencing [91]. Emu utilizes an expectation-maximization approach, which enables precise and reliable taxonomic assignments, minimizing false positives and negatives [91].

To further refine taxonomic assignment, a customized 16S rRNA database was constructed by combining reference databases with sequences derived from previous studies. A minimum abundance threshold of 0.01 was established to exclude low-representative taxa or potential artifacts. Classified reads were grouped into operational taxonomic units (OTUs) at a 97% similarity threshold, which enabled the operational definition of the different bacterial species present in the samples.

### 2.5. Community Profiling and Statistical Testing

The resulting OTU table was analyzed using the Microbiome Analyst software version 2.0 to obtain a comprehensive overview of the microbial community structure and diversity. Initially, singleton OTUs—those present in only one sample—were removed to reduce noise and enhance the robustness of the analyses. Subsequently, a logarithmic transformation was applied to the abundance data to normalize the distribution and improve the interpretability of the results.

Principal Coordinates Analysis (PCoA) was performed based on a Bray-Curtis distance matrix to assess differences in the microbial community composition across sample groups. This analysis facilitated the visualization of sample relationships and the identification of clusters with similar microbial compositions. In addition, an Analysis of Similarities (ANOSIM) was conducted to determine whether statistically significant differences existed in community structure between the compared groups.

Furthermore, a rarefaction curve was constructed using the Vegan package in R to evaluate sampling coverage and microbial community richness [92]. This curve assessed whether the number of sequences obtained was sufficient to capture the total community diversity and if there were differences in richness between the sample groups.

### 2.6. Data Processing and Heat Tree Visualization of Microbial Communities

The heat tree analysis was performed using the R programming language (version 4.3.3) and the integrated development environment RStudio. Several R libraries were utilized, including Metacoder for hierarchical taxonomic data analysis and visualization, Dplyr for efficient data manipulation, and Vegan for ecological diversity analysis [92,93]. These tools were selected for their robustness and widespread use in microbiota data analysis.

The microbiota dataset used in this study was derived from 16S rRNA sequencing data processed through the MicrobiomeAnalyst pipeline [94]. The dataset comprised two primary files: one containing taxonomic read abundance per sample and another with metadata detailing sample attributes, such as experimental treatments and tissue sources. Both files were imported into R, and an initial exploratory analysis was conducted to assess data integrity and structure.

A rigorous filtering process was implemented to ensure data reliability and mitigate the influence of sequencing artifacts. Taxa with low read counts, specifically those with fewer than five reads, were removed to minimize the impact of sequencing errors. Furthermore, taxa exhibiting zero abundance across all samples were excluded. A prevalence threshold of 20% was applied, ensuring that only taxa present in at least 20% of the samples were retained. This filtering process was conducted separately for tissue types, including gills and digestive glands, for potential tissue-specific variations in microbial communities.

The heat tree visualization was generated using the Metacoder package, which enables the hierarchical representation of microbial communities [95]. In this visualization, node size corresponds to taxon abundance, while node color indicates statistical differences across experimental conditions. Prior to visualization, the dataset was transformed into a format compatible with the Metacoder package. Taxonomic hierarchies were structured according to lineage, and a taxmap object was created to organize and analyze taxonomic relationships.

### 2.7. Linear Discriminant Analysis Effect Size (LEfSe) and Correlation Network Analysis

The linear discriminant analysis effect size (LEfSe) method was employed to identify significant differences in bacterial species abundance between gill samples from challenged and controlled individuals. A significance threshold of FDR-adjusted *p*-value < 0.05 and a logarithmic, linear discriminant analysis (LDA) score ≥ 4.0 were set as cut-off values to identify differentially abundant taxa. The top 15 discriminative features were visualized using a dot plot highlighting the primary bacterial taxa driving the differences between groups.

To further explore microbial interactions, a network correlation analysis was performed to evaluate the co-occurrence patterns of microbial taxa in the samples. Networks were constructed using the Sparse Correlations for Compositional Data (SparCC) algorithm, which is particularly suited for microbiota data due to its ability to handle compositional structures. The correlation network was estimated using 100 bootstrap permutations, with a significance threshold of *p* < 0.05 and a minimum correlation coefficient of 0.3. These parameters were chosen to ensure the identified associations’ robustness and minimize the inclusion of spurious correlations. This approach helps identify potential interactions and dependencies among bacterial species within the microbiota.

### 2.8. Prediction of Metagenomic Functional Potential

PICRUSt2 (Phylogenetic Investigation of Communities by Reconstruction of Unobserved States) was employed to infer the functional potential of the microbiota in the gill and digestive gland tissues of *M. chilensis* under hypoxic conditions based on marker gene sequences [96]. The R package ggpicrust2 was used to facilitate the functional analysis and visualization [97]. Given that PICRUSt2 is optimized for short-read sequencing data, a preprocessing step was necessary to adapt our long-read amplicon sequences generated through nanopore technology. The software HyperEx v0.1.0 extracted the V3–V4 regions from the full-length 16S rRNA sequences, generating a FASTA file suitable for downstream processing.

The resulting FASTA and BIOM files generated from the operational taxonomic unit (OTU) table were used as input for PICRUSt2 analysis. The functional pathways were constructed using the MetaCyc database to provide a comprehensive functional profile of the microbial communities [98]. The relative abundances of pathways were visualized using the R software version 4.3.3. Statistical Analysis of Metagenomic Profiles (STAMP) was employed [99] to test for significant differences in pathway contributions between groups. A chi-square test corrected with the Benjamini–Hochberg false discovery rate (FDR) was applied to control for multiple comparisons, with a significance threshold set at an FDR-adjusted *p*-value of <0.05.

For visualization purposes, the final plots included only data with a minimum relative abundance of 0.5% to focus on the most relevant functional changes. This approach enabled a detailed evaluation of pathway-level functional shifts associated with the microbiota’s response to hypoxic stress, providing insights into the potential functional adaptations within the microbial community.

### 2.9. Data Availability

The nanopore data for DNA analysis were deposited in the National Center for Biotechnology Information Short Reads Archive (NCBI-SRA) under the BioProject accession number PRJNA1240298.

## 3. Results

### 3.1. Alpha and Beta Diversity Analysis of M. chilensis Microbiota Under Normoxia and Hypoxia

Alpha and beta diversity analyses of the *M. chilensis* microbiota under normoxic and hypoxic conditions revealed significant differences in microbial composition and structure across both studied tissues (gills and digestive gland) (Figure 1). Rarefaction curves (Figure 1A) showed an apparent decrease in microbial diversity under hypoxia compared to normoxia, particularly pronounced in the gills. The digestive gland showed lower diversity only during the first hypoxic sampling. Principal Coordinate Analysis (PCoA) (Figure 1B) revealed a distinct clustering of normoxic and hypoxic samples for both tissues, indicating substantial shifts in microbial community composition. Alpha diversity indices (Figure 1C) confirmed a significant decrease in microbial diversity in both tissues under hypoxia. This decline was more pronounced in the gills, suggesting a greater sensitivity of the microbial community present to low oxygen compared to the digestive gland.

### 3.2. Taxonomic Shifts in the Microbiota of M. chilensis Under Normoxia and Hypoxia

Figure 2 presents a heat tree analysis, where branch color reflects the log2 of the mean ratio between bacterial taxa’s relative abundance under normoxia (blue) and hypoxia (red). This approach visualizes taxonomic changes across hierarchical levels, highlighting variations induced by oxygen conditions.

In gills, an apparent decrease in the relative abundance of specific taxa under hypoxia was observed, while others exhibited an increase. This difference is represented by branch color: taxa with decreased abundance under hypoxia are blue, and those with increased abundance are red. Dominant taxa under normoxia included Roseobacteraceae, *Oceanicola*, *Roseobacter*, *Thalassobius*, *Phaeobacter*, Octadecabacter, *Sulfitobacter*, *Leisingera*, *Ruegeria*, *Loktanella*, *Tateyamaria*, *Roseovarius*, *Litoreibacter*, *Pelagimonas*, *Pacifibacter*, *Shimia*, *Actibacterium*, *Saggitula*, Rhodobacteraceae, *Aliiroseovarius*, *Yoonia*, *Pseudophaeobacter*, *Planktotalea*, *Sedimentitalea*, *Amylibacter*, *Aestuariibius*, and *Neptunicoccus*. Conversely, taxa representative of hypoxia included Campylobacterales, *Poseidonibacter*, Arcobacteraceae, Campylobacteraceae, Campylobacter, *Aliarcobacter*, *Arcobacter*, *Malaciobacter*, *Halarcobacter*, Thiovulaceae, *Elizabethkingia*, *Weeksellaceae*, *Saccharicrinis*, *Paludibacter*, *Crocinitomix*, Crocinitomicaceae, *Labilibacter*, Prolixibacteraceae, *Marinifilum*, *Olleya*, *Antarticcibacterium*, Firmicutes, and Bacillales.

The digestive gland displayed a similar response, with differential patterns in taxonomic abundance under hypoxia. Hypoxia significantly altered the microbiota composition, with several taxa exhibiting notable changes in relative abundance. Dominant taxa under normoxia included *Octadecabacter*, *Sulfitobacter*, *Thalassobius*, *Phaeobacter*, *Roseobacter*, *Leisingera*, *Antarctobacter*, *Roseovarius*, *Tateyamaria*, *Shimia*, *Pacificibacter*, *Litoreibacter*, *Pelagimonas*, *Aliiroseovarius*, *Amylibacter*, *Sedimentitalea*, *Planktotalea*, Rhodobacteraceae, Lacipirellulaceae, *Polaribacter*, and *Aquimarina*. In contrast, taxa enriched under hypoxia were *Acidovorax*, *Delftia*, *Curvibacter*, *Burkholderia*, *Cupriavidus*, *Paraburkholderia*, *Neisseria*, *Massilia*, *Desulfovibrio*, *Solidesulfovibrio*, *Pseudodesulfovibrio*, *Acidithiobacillus*, Acidiferrobacteraceae, Nevskiales, Steroidobacteraceae, Ectothiorhodospiraceae, *Marinobacterium*, *Pseudoalteromonas*, *Shewanella*, *Vibrio*, *Francisella*, *Yersinia*, *Serratia*, Pectobacteriaceae, Enterobacteriaceae, *Ancylomarina*, Marinifilaceae, Marinilabiliales, Bacteroidales, and Flavobacteriales.

### 3.3. Analysis of Bacterial Genus Relative Abundance in the Microbiota of M. chilensis Under Normoxia and Hypoxia

Heatmap analysis revealed distinct shifts in the relative abundance of bacterial genera in the gill and digestive gland microbiotas of *M. chilensis* under normoxic and hypoxic conditions (Figure 3 and Figure 4). The microbial communities clustered into two distinct groups in both tissues, reflecting differential responses to oxygen availability. Figure 3A and Figure 4A illustrate these patterns, with normoxic (blue) and hypoxic (red) conditions highlighting specific genera that were differentially distributed, suggesting a tissue-specific microbial adaptation to hypoxia.

Cluster 1 consisted of bacterial genera that showed a marked decrease in abundance under hypoxic conditions. In the gills (Figure 3B), these genera exhibited a significant reduction in relative abundance during hypoxia exposure, with a similar trend observed in the digestive gland (Figure 4B). Representative genera in the gill cluster included *Salmonella*, *Thalassobius*, *Roseobacter*, *Cocleimonas*, *Nitratireductor*, *Planktomarina*, *Marinicella*, *Yoonia*, *Neptunicoccus*, *Tenacibaculum*, *Cellulophaga*, *Leucothrix*, *Tritonibacter*, *Jannaschia*, *Lacinutrix*, *Boseongicola*, *Sedimentitalea*, *Pseudahrensia*, *Pseudoruegeria*, and *Phaeobacter*. In the digestive gland, prominent genera included *Thiomicrorhabdus*, *Mobilisporobacter*, *Klebsiella*, *Helicobacter*, *Spongiibacter*, *Paraglaciecola*, *Citrobacter*, *Shewanella*, *Cellvibrio*, *Vibrio*, *Solidesulfovibrio*, *Rheinheimera*, *Serratia*, *Aquella*, *Glaciecola*, *Franconibacter*, *Latilactobacillus*, *Oceaniserpentilla*, *Lacticaseibacillus*, and *Labilibaculum*.

In contrast, Cluster 2 comprised bacterial genera that increased in abundance under hypoxia. In the gills (Figure 3C), these genera showed a significant rise in relative abundance in response to hypoxic exposure, and a similar pattern was evident in the digestive gland (Figure 4C). The predominant gill genera under hypoxia included *Poseidonibacter*, *Arcobacter*, *Phocaeicola*, *Marinifilum*, *Halarcobacter*, *Methylothermus*, *Azospirillum*, *Labilibaculum*, *Bacteroides*, *Saccharicrinis*, *Francisella*, *Salegentibacter*, *Draconibacterium*, *Paludibacter*, *Elizabethkingia*, *Polaribacter*, *Pedobacter*, *Malaciobacter*, *Roseimarinus*, and *Aliarcobacter*. The digestive gland featured hypoxia-associated genera such as *Sideroxydans*, *Planktotalea*, *Marinicella*, *Nitratireductor*, *Mariniblastus*, *Ahrensia*, *Litoreibacter*, *Profundibacter*, *Pseudahrensia*, *Seohaeicola*, *Jannaschia*, *Pelagimonas*, *Lentibacter*, *Olleya*, *Thalassobius*, *Octadecabacter*, *Sulfitobacter*, *Amylibacter*, *Fuerstiella*, and *Aliiroseovarius*.

### 3.4. Linear Discriminant Analysis

Linear Discriminant Analysis (LDA) demonstrated distinct bacterial community compositions in the gills and digestive gland of *M. chilensis* under normoxic and hypoxic conditions (Figure 5).

In the gills (Figure 5A), 31 bacterial species were significantly more abundant under normoxia, including *Aquimarina macrocephali*, *Aquimarina muelleri*, and *Flavobacteriaceae bacterium*. Conversely, 19 species exhibited increased abundance under hypoxia, such as *Poseidonibacter parvus*, *Poseidonibacter lekithochrous*, and *Arcobacter nitrofigilis*.

Similarly, the digestive gland (Figure 5B) displayed 14 bacterial species enriched under normoxia, including *Polaribacter* sp. *ALD11*, *Polaribacter* sp. *BM10*, and *Plaribacter atrinae*. In contrast, six species showed increased abundance under hypoxia, including *Francisella tularensis*, *Citrobacter freundii*, and *Shigella sonnei*.

### 3.5. Functional Potential Prediction of the M. chilensis Microbiota Under Normoxia and Hypoxia

**Figure 6:**
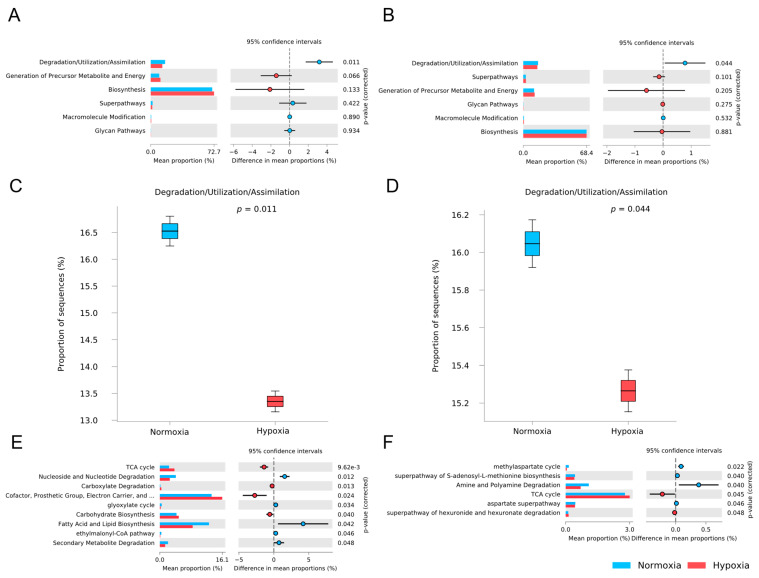
Prediction of the functional potential of the microbiota from gills and digestive gland of *M. chilensis* based on Picrust2. Bars represent the mean proportions of microbiota pathways in normoxia (blue) and hypoxia (red) individuals. The differences between the conditions for the Metacyc top level are presented for gills (**A**) and digestive gland (**B**) with their respective corrected *p*-values. (**C**,**D**) show the specific values for the degradation/utilization/assimilation pathways for gills and digestive gland, respectively. Finally, the Metacyc secondary levels for degradation/utilization/assimilation with significant differences (corrected *p*-value < 0.05) within experimental conditions are also presented for gills (**E**) and digestive gland (**F**).

Picrust2 analysis revealed alterations in the functional potential of the *M. chilensis* microbiota between normoxia and hypoxia conditions (Figure 6). Among the metabolic pathways analyzed, only the degradation/utilization/assimilation category showed a significant difference between conditions (Figure 6A,B). Notably, both tissues exhibited a higher proportion of sequences assigned to degradation/utilization/assimilation pathways under normoxia compared to hypoxia (Figure 6C,D).

The metabolic pathways enriched under normoxia and hypoxia differed between the gills and digestive gland (Figure 6E,F). All degradation/utilization/assimilation pathways were different for both tissues, except for the TCA cycle which was more enriched than other pathways in the digestive gland. In the gills, normoxia favored pathways related to cofactor, prosthetic group, and electron carrier biosynthesis, as well as fatty acid, lipid, and carbohydrate biosynthesis, whereas the digestive gland exhibited a predominance of the TCA cycle, amine and polyamine degradation, and aspartate metabolism. Under hypoxia (Figure 6E), the gills showed an increase in cofactor, prosthetic group, electron carrier biosynthesis, carbohydrate biosynthesis, and the TCA cycle, accompanied by a decline in fatty acid and lipid biosynthesis, nucleoside and nucleotide degradation, and secondary metabolite degradation. In the digestive gland (Figure 6F), hypoxia induced an upregulation of the TCA cycle and hexuronide and hexuronate degradation, while pathways related to amine and polyamine degradation, aspartate metabolism, S-adenosyl-L-methionine biosynthesis, and the methylaspartate cycle were downregulated.

### 3.6. Dynamics of Bacterial Pathogens

**Figure 7:**
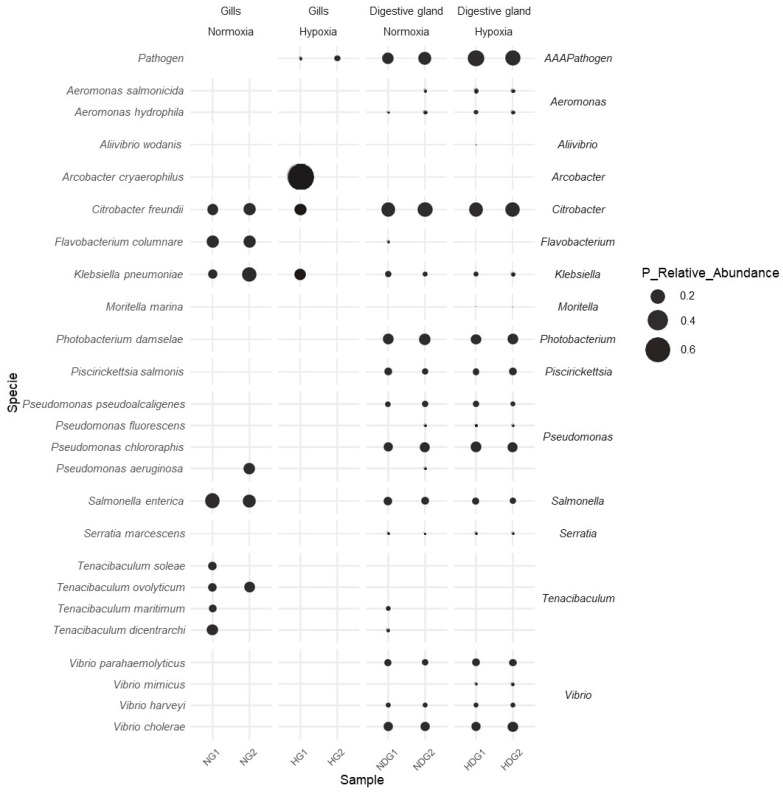
Dot plot showing the dynamics in the relative abundance of fish bacterial pathogens associated with gills and digestive glands of *M. chilensis* exposed to different oxygenation levels.

We evaluated the presence and abundance of specific groups to explore whether changes in the microbiota due to hypoxia lead to an increase in the community of pathogenic bacteria. The objective was to assess whether hypoxia promotes bivalves as reservoirs for aquatic pathogens. Figure 7 presents a scatter plot illustrating the dynamics in the relative abundance of various fish bacterial pathogens in *M. chilensis* under normoxic and hypoxic conditions. Each point on the graph represents the relative abundance of a specific pathogen at different levels of oxygen. 

In the gills of *M. chilensis*, an increase in the relative abundance of some bacterial pathogens was observed under hypoxic conditions compared to normoxia. Pathogens that showed a significant increase included *Arcobacter cryaerophilus*, with smaller increases seen in *Citrobacter freundii* and *Klebsiella pneumoniae*. Conversely, some bacterial pathogens in the gills of *M. chilensis* disappeared under hypoxic conditions compared to normoxia. These pathogens included *Flavobacterium columnare*, *Salmonella enterica*, and *Tenacibaculum ovolyticum*.

Similarly, the digestive gland of *M. chilensis* also experienced an increase in the relative abundance of several bacterial pathogens under hypoxia. Pathogens that showed a notable increase included *Aliivibrio wodanis*, *Pseudomonas fluorescens*, *Moritella marina*, and *Vibrio mimicus*. Additionally, in the digestive gland of *M. chilensis*, several bacterial pathogens disappeared under hypoxic conditions compared to normoxia. Pathogens that disappeared included *Flavobacterium columnare*, *Tenacibaculum maritimum*, and *Tenacibaculum dicentrarchi*. Moreover, a reduction in the relative abundance of *Vibrio cholerae*, *Pseudomonas chlororaphis*, and *Citrobacter freundii* was also observed.

## 4. Discussion

This study highlights the importance of conducting controlled hypoxia exposure experiments to understand and predict changes in the microbiota of bivalve mollusks, such as *M. chilensis*, under environmental stress associated with climate change [15]. Hypoxia, regarded as a significant environmental stressor, interacts in complex ways with other environmental factors, significantly affecting the health and performance of marine organisms [100,101]. Advancing our understanding of the microbiota’s role in the physiological response of organisms to these stressors has become essential, as it can reveal critical mechanisms of adaptation and resilience [30,102,103,104]. Expanding on previous studies, this work focused on evaluating the impact of hypoxia on the gill and digestive gland microbiota of *M. chilensis* [15,105,106].

In this context, our study is pioneering in demonstrating the effects of oceanic hypoxia on the gill and digestive gland microbiota of mussels at the species level. The gills, in addition to their respiratory function, play a key role in nutrition and immune defense, hosting symbiotic microbial communities that contribute to carbon and nitrogen fixation [107,108,109,110]. Our results indicate that hypoxia induces profound changes in both the composition and function of the microbiota in these tissues, primarily by eliminating bacterial groups unable to tolerate low oxygen conditions. The observed restructuring of the microbiota under hypoxia suggests adaptive and selective effects, reinforcing the microbiota’s ability to respond to environmental stress. These changes likely result from selective pressure favoring bacterial communities with greater tolerance to hypoxic conditions, possibly mediated by adaptive mechanisms such as quorum sensing, which regulate colonization, virulence, and stress resistance in oxygen-depleted environments [27,28,108,111,112,113,114,115]. However, this adaptive capacity also presents potential risks. The selective pressure exerted by hypoxia may facilitate the proliferation of opportunistic pathogens, potentially compromising the host’s immune system and its ability to resist infections and other environmental stressors. Our results provide evidence of this selective pressure, manifested in a reduction of bacterial species richness and diversity, particularly in the gills, aligning with responses observed in other aquatic ecosystems [1,116,117]. Functional changes in the microbiota of *M. chilensis*, especially in lipid and fatty acid biosynthesis pathways, suggest metabolic adaptations to hypoxia. These findings corroborate previous studies in other bivalves [15]. These changes are likely strategies to optimize energy efficiency and minimize biomass production in resource-limited environments [16,118].

Alterations in the functionality of the microbiota have direct implications for host physiology [119]. Reducing the production of essential metabolites, such as vitamins and amino acids, may affect the nutrition and overall health of *M. chilensis* [120]. Additionally, the decrease in microbial diversity and functionality could limit the microbiota’s ability to contribute to critical metabolic processes, such as nutrient digestion and the production of immunomodulatory compounds [121]. The results reveal a decrease in the activity of degradation, utilization, and nutrient assimilation pathways in the digestive gland under hypoxic conditions. This phenomenon could affect essential cellular processes, such as proliferation and differentiation, limiting the mussel’s growth and adaptive capacity in response to additional stress factors [122,123]. The complex interaction between biosynthesis, metabolite generation, and degradation pathways under hypoxia indicates a co-evolutionary adaptation process between *M. chilensis* and its microbiota. This symbiotic relationship is crucial for maintaining host homeostasis; any disruption in this balance may lead to dysbiosis, increasing susceptibility to opportunistic or polymicrobial infections. In aquaculture and marine environments, dysbiosis has been associated with mass mortality events and disease outbreaks, underscoring the importance of the microbiota in host health [124].

The variation in the intestinal microbiota composition of *Mytilus* across individuals and populations reflects the influence of diet, host genetics, and environmental conditions [125,126,127,128,129,130]. Aquaculture practices also shape this microbial structure, as seen in the differences between the microbiota of farmed and wild mussels [131]. In the gills of *M. chilensis*, the increased presence of bacterial groups associated with hypoxic environments is a clear consequence of the selective pressure of low oxygen availability. These adaptations may involve the production of antioxidants and the utilization of alternative metabolic pathways, indicating a complex metabolic interaction between the bacteria and the host [132,133].

The dominant microbiota in the digestive gland of *M. chilensis* includes bacteria from the phyla Actinobacteria, Proteobacteria, Bacteroidetes, and Firmicutes, consistent with other mussel species and aquatic organisms [15,44,134,135,136,137,138,139,140,141,142,143,144,145,146,147,148]. In contrast, the gill microbiota is dominated by Proteobacteria and Firmicutes, suggesting specialized and complementary roles in mussel physiology. Our findings are consistent with previous studies in other aquatic environments [136]. The prevalence of Bacteroidetes and Firmicutes in the digestive tract indicates a specialized symbiotic relationship with an enzymatic arsenal for degrading complex polysaccharides, such as cellulose and chitin, found in plankton and marine detritus [135,149,150,151]. These bacteria produce various bioactive compounds, such as antibiotics and pigments, which can benefit both the host and the microbiota [152]. Antibiotics may protect the host from pathogens, while pigments may function as antioxidants, reducing oxidative stress [153,154]. Firmicutes, with their ability to form spores, contribute to the stability of the intestinal microbiota under stressful conditions [155,156,157]. Additionally, some Firmicutes produce short-chain fatty acids as fermentation byproducts, which have anti-inflammatory properties and modulate the host’s immune response [158].

Hypoxia significantly altered the microbial community structure of *M. chilensis*, favoring facultative anaerobes and opportunistic pathogens while reducing beneficial microbial functions. Proteobacteria in the gills of *M. chilensis* participate in the degradation of organic matter in marine environments [159,160,161,162]. Their metabolic versatility allows them to utilize a wide range of organic compounds, significantly contributing to nutrient cycling [163,164,165,166,167,168]. However, a shift toward *Vibrio*, *Aeromonas*, and *Desulfovibrio* was observed under hypoxic conditions, indicating an increased risk of pathogenicity and altered sulfur metabolism [169,170,171]. The selective pressure exerted by low oxygen availability may enhance the survival of bacteria capable of utilizing alternative electron acceptors, potentially increasing oxidative stress in the host and disrupting metabolic homeostasis [172,173,174,175,176].

The study of the microbiota associated with *M. chilensis* reveals the dominance of bacteria belonging to the classes Alphaproteobacteria, Bacteroidia, Epsilonproteobacteria, and Gammaproteobacteria in the gills and digestive gland. These classes exhibit diverse and ecologically relevant metabolic functions in aquatic ecosystems, participating in biogeochemical and ecological cycles [167,177,178,179,180,181]. The presence of Epsilonproteobacteria in the gills suggests an adaptation to sulfuric niches and a potential symbiotic role in the oxidation of reduced compounds [182,183]. Alphaproteobacteria and Gammaproteobacteria are key components of the *M. chilensis* microbiota, particularly under normoxic conditions. Their abundance may correlate with water quality, serving as potential bioindicators of dissolved organic matter from anthropogenic sources [184,185]. Gammaproteobacteria play a crucial role in the degradation of complex organic compounds, contributing to nutrient biogeochemical cycles [186,187,188]. The order Rhodobacterales, within the class Alphaproteobacteria, dominates the gill and digestive microbiota of *M. chilensis*. These bacteria break down organic matter, facilitating the host’s nutrient absorption [181]. Furthermore, they may modulate the immune response, enhancing the mussel’s survival in challenging environments [189,190]. A significant aspect is the Roseobacteraceae family’s sensitivity to hypoxia. The reduction of these bacteria under low oxygen conditions suggests a disruption in microbial trophic networks, potentially affecting host health [191,192,193].

The taxonomic analysis of the digestive gland reveals the dominant presence of bacterial genera *Shewanella*, *Aeromonas*, and *Vibrio* as key components of the core bacterial community in the digestive gland. These bacteria are known for their pathogenic potential and ability to act as reservoirs of plasmids encoding antibiotic resistance genes [194,195,196]. The horizontal transfer of these genes, facilitated by conjugation, transformation, and transduction mechanisms, increases the risk of antimicrobial resistance dissemination, posing a significant public health concern [197,198,199,200,201,202]. This phenomenon is not limited to clinical settings but has also been documented in natural aquatic ecosystems, suggesting that these environments can serve as global reservoirs of resistance genes [203,204,205].

The increasing antimicrobial resistance, exacerbated by climate change and the indiscriminate use of antibiotics in aquaculture, highlights the urgent need to develop more effective control strategies [206,207,208,209,210,211]. Under hypoxic conditions, a reduction in the populations of *Shewanella* and *Vibrio* was observed, while *Aeromonas* demonstrated a remarkable ability to adapt and maintain its presence. This adaptability may be related to its diverse genetic repertoire, which includes virulence factors and the ability to form biofilms [212,213,214,215,216,217].

Pathogenic species such as *Aeromonas hydrophila* and *Aeromonas salmonicida* cause significant losses in aquaculture and pose a global health threat due to their ability to acquire antibiotic resistance genes [201,206,218,219,220,221,222,223,224,225,226,227]. The coevolution between Aeromonas and hosts such as *M. chilensis* suggests a symbiotic relationship in which the bacteria may facilitate digestion and offer protection against other pathogens [228,229,230,231,232,233,234,235,236,237,238,239,240,241,242,243,244,245,246]. However, this symbiosis can be disrupted under environmental stress, such as hypoxia, promoting Aeromonas as an opportunistic pathogen.

The decrease in *Shewanella* under hypoxic conditions is noteworthy, considering its ability to utilize nitrate as an electron acceptor in anaerobic environments [247,248,249]. This reduction in abundance could be due to competition for nutrients or the production of antimicrobial compounds by other microbial species’. The presence of *Vibrio* in the core microbiota of *M. chilensis* suggests a potential mutualistic relationship, which is consistent with other studies [240,250,251]. However, some *Vibrio* species are pathogenic to both bivalves and humans [250,252,253,254,255,256,257]. Although *Vibrio* demonstrates ecological adaptability, its dependence on oxygen reduces its prevalence under hypoxic conditions. This decrease may benefit the health of *M. chilensis* by reducing *Vibrio*-induced diseases, but it could also increase vulnerability to other pathogenic species such as *Vibrio mimicus*. This pathogen has been associated with disease outbreaks in various bivalve species, causing high mortality rates and significant economic losses in aquaculture [258]. *V. mimicus* thrives in hypoxic conditions and poses a threat due to biofilm formation, antibiotic resistance, and zoonotic potential [259,260]. An increase in the genus *Acinetobacter* was observed in the digestive gland under hypoxic conditions. This genus is notable for its ability to persist in diverse environments and facilitate horizontal gene transfer, including those conferring resistance to multiple clinically relevant antibiotic classes. Pathogenic strains of *Acinetobacter*, characterized by their multidrug resistance, represent a critical public health threat in both clinical settings and natural ecosystems [222].

Our findings confirm the absence of *Psychrilyobacter* in farmed *M. chilensis* compared to wild populations, indicating that habitat characteristics significantly influence microbial composition [131]. Anaerobic conditions in natural ecosystems support *Psychrilyobacter* colonization, whereas suspended aquaculture systems limit such environments [163,261,262,263,264]. Reduced microbial diversity in aquaculture could impair mussel adaptation to environmental changes and increase susceptibility to pathogens over time [265]. The presence of *Aquimarina macrocephali* in the gills of *M. chilensis* under normoxic conditions suggests an adaptation to this oxygen-rich environment. This is supported by its enzymatic profile for reducing oxidative stress and its potential role in the degradation of organic matter [266]. This bacterium may contribute to the cleaning of gill surfaces, facilitating nutrient acquisition and enhancing host health. Additionally, its ability to degrade chitin suggests a possible symbiotic interaction with the mussel, as chitin is a common structural component in plankton and microorganisms that form part of the bivalve diet [151,266]. However, *A. macrocephali*’s resistance to multiple antibiotics raises concerns about its role as a reservoir of resistance genes, potentially facilitating the spread of these genetic elements within the marine ecosystem and the food chain [266,267,268,269,270]. Antibiotic resistance in *A. macrocephali* could be linked to acquiring plasmids and exposure to subtherapeutic antibiotic doses in the aquatic environment, stemming from aquaculture and wastewater [271,272].

The comprehensive integration of transcriptomic and microbiota profiling in *M. chilensis* exposed to hypoxic stress has revealed a tightly interconnected network of metabolic reprogramming, immune modulation, and microbial community restructuring, underscoring the profound physiological consequences of oxygen deprivation. Hypoxia-induced metabolic shifts, characterized by the suppression of aerobic respiration and the upregulation of anaerobic pathways, were accompanied by significant alterations in the microbiota, favoring facultative anaerobes and opportunistic pathogens, thereby indicating microbial dysbiosis that could compromise host metabolic efficiency and overall homeostasis. Concurrently, immunosuppression was evident through the downregulation of immune-related genes, increased oxidative stress marked by elevated reactive oxygen species (ROS) levels, and a greater abundance of pathogenic taxa, collectively suggesting heightened host vulnerability to infections. The response exhibited tissue specificity, with gill tissues displaying more pronounced transcriptomic and microbial shifts than the digestive gland, potentially engaging compensatory metabolic pathways to mitigate hypoxia-induced stress [77].

A more personalized approach will be required to effectively manipulate immune functions through microbiota-based therapies, one that identifies specific microorganism-host relationships [104]. In the context of bivalves, high-throughput sequencing has been instrumental in managing diseases in aquaculture [273,274]. Prebiotics and probiotics in mussel farming offer a promising approach to enhancing larval resistance to adverse effects such as climate change [273,275]. These adaptations could support the survival of populations in an evolving marine environment. Early colonization by a complex microbiota or specific symbionts can induce lasting epigenetic modifications, promoting protective immunity and greater resilience to environmental stressors associated with climate change [276,277,278]. These early epigenetic alterations can have long-term protective effects, reducing the risk of diseases in later life stages and enhancing the organism’s adaptive capacity [276,277,278]. Additionally, molecular research on the interactions between hypoxia and other environmental factors, such as temperature, pH, and salinity, is crucial for managing the impacts of hypoxia on aquatic ecosystems [274,279]. Monitoring oxygenation conditions in marine habitats will be essential to maintaining the health and robustness of key species. Identifying bacterial genera sensitive or resistant to hypoxia will provide critical insights for developing sustainable management strategies.

An integrated approach based on the hologenome concept is recommended. This approach includes developing predictive models that incorporate environmental, microbial, and host factors to better understand and manage the effects of hypoxia in these ecosystems. Moreover, expanding studies on the *M. chilensis* hologenome, which includes viruses and microeukaryotes whose identities and functions are still in the early stages of research, is necessary [280]. Implementing sustainable management practices, such as reducing eutrophication and restoring coastal habitats, could improve water quality and protect marine biodiversity.

## 5. Conclusions

This study represents the first comprehensive report on the effects of hypoxia, a global environmental concern, on the gills and digestive glands of the mussel *M. chilensis*. Our findings show that hypoxia significantly affects the structure, relative abundance, community composition, species richness, and diversity of microbial communities associated with these tissues. The changes in the microbiota induced by hypoxia have substantial implications for mussel physiology, influencing essential processes such as digestion, nutrient absorption, and immune response. Additionally, our results suggest that hypoxia encourages the growth of opportunistic and pathogenic bacteria, underscoring the need for further investigation into the underlying mechanisms of these changes and their functional implications for the organism. Thus, maintaining optimal dissolved oxygen levels in aquaculture systems is crucial for preserving the health of *M. chilensis* and ensuring the sustainability of aquaculture practices. Future research is necessary to develop mitigation strategies that can reduce the negative impacts of this stressor on aquatic ecosystems.

## Data Availability

The original contributions presented in the study are included in the article; further inquiries can be directed to the corresponding authors.

## Abbreviations

The following abbreviations are used in this manuscript:

°C	grados Celsius
ANID	Agencia Nacional de Investigación y Desarrollo
ANOSIM	Analysis of Similarities
CA	California
CEBB	Ethics Committee of the Universidad de Concepción
C.G.-E.	Cristian Gallardo Escárate
D.V.-M	Diego Valenzuela Miranda
DO	Dissolved oxygen
FDR	false discovery rate
FONDAP	Fondo de Financiamiento de Centros de Investigación en Áreas Prioritarias
INCAR	Interdisciplinary Center for Aquaculture Research
IPIAP	Instituto Público de Investigación de Acuicultura
LDA	linear discriminant analysis
LDOW	low dissolved oxygen water
LEfSe	Linear Discriminant Analysis Effect Size
log_2_	logarithm base 2
*M. chilensis*	Mytilus chilensis
MA	Massachusetts
MDPI	Multidisciplinary Digital Publishing Institute
mg/L	milligrams per liter
M.M.-R	Milton Montúfar Romero
M.F.M.-R	María Fernanda Morales-Rivera
n	sample size
NCBI	National Center for Biotechnology Information
OTUs	operational taxonomic units
PCoA	Principal Coordinates Analysis
PCR	Polymerase Chain Reaction
pH	potential of hydrogen
PICRUSt2	Phylogenetic Investigation of Communities by Reconstruction of Unobserved States
Q-score	Quality score
rRNA	ribosomal ribonucleic acid
SENESCYT	Secretaría de Educación Superior, Ciencia, Tecnología e Innovación
SparCC	Sparse Correlations for Compositional Data
SRA	Sequence Read Archive
STAMP	Statistical Analysis of Metagenomic Profiles
TCA	Tricarboxylic Acid Cycle
UK	United Kingdom
USA	United States of America
V.V.-M	Valentina Valenzuela-Muñoz
